# Effects of early versus delayed terlipressin infusion on hemodynamics and catecholamine requirements in ovine septic shock

**DOI:** 10.1186/cc9513

**Published:** 2011-03-11

**Authors:** TG Kampmeier, M Westphal, S Rehberg, A Morelli, M Lange, H Van Aken, C Ertmer

**Affiliations:** 1 University Hospital of Münster, Germany; 2 University of Rome 'La Sapienza', Rome, Italy

## Introduction

Terlipressin (TP) is increasingly used in catecholamine-dependent septic shock. Whereas recent data suggest advantages of continuous infusion over repetitive bolus infusion, the optimal time of TP initiation remains unclear. The present study was designed as a prospective laboratory experiment to compare the impact of early versus delayed TP infusion on key hemodynamic variables, as well as fluid and catecholamine requirements in ovine septic shock.

## Methods

Twenty-three healthy female sheep were anesthetized and instrumented for hemodynamic monitoring. A median laparotomy was performed and 1.5 g/kg feces were taken from a cecal incision. After gut suture and insertion of peritoneal drains, the abdomen was closed. Following baseline measurements, autologous feces were injected into the abdominal cavity via a drain. When septic shock had established (MAP <60 mmHg and arterial lactate >1.8 mmol/l), causal therapy (meropenem infusion and peritoneal lavage every 8 hours) and supportive treatment (volume therapy guided by stroke volume variation and global end-diastolic volume, as well as norepinephrine infusion to maintain MAP >60 mmHg) were initiated. Sheep were randomized to placebo (*n *= 7), or to continuous TP infusion (2 μg/kg/hour) started at shock onset (early TP; *n *= 8), or to continuous TP infusion (2 μg/kg/hour) started when NE requirements exceeded 0.3 μg/kg/minute (delayed TP; *n *= 8). After 24 hours of therapy, the surviving sheep were killed in deep anesthesia.

## Results

Whereas two out of seven sheep allocated to the placebo group survived, three out of eight survived in both TP groups. Whereas hemodynamic variables were similar among groups, cumulative open-label NE requirements were significantly lower in the early TP group (0.8 ± 0.6 mg/kg) as compared with both the placebo group (2.7 ± 0.6 mg/kg) or the delayed TP group (2.2 ± 0.5 mg/kg; each *P *< 0.05). Total fluid requirements and increase in body weight tended to be lower in the early TP group as compared with the other two groups.

## Conclusions

Early TP infusion reduces catecholamine and fluid requirements as compared with delayed TP therapy and placebo in ovine septic shock.

